# Assessing determinants of the availability of HIV tracer commodities in health facilities in Wakiso District, Uganda

**DOI:** 10.1080/20523211.2024.2306846

**Published:** 2024-02-07

**Authors:** Falisy Lule, Kalid Rajab, Stany Banzimana, Domina Asingizwe

**Affiliations:** a EAC Regional Centre of Excellence for Vaccines, Immunization and Health Supply Chain Management, College of Medicine and Health Sciences, University of Rwanda, Kigali, Rwanda; b School of Pharmacy, College of Health Science, Makerere University, Kampala, Uganda

**Keywords:** HIV/AIDS tracer commodities, availability, stock outs, Wakiso District-Uganda

## Abstract

**Background:**

HIV/AIDS commodity stock-outs are still rampant in most African Countries causing treatment interruption, antiretroviral resistance, treatment failure, morbidity and mortality. Therefore, this study aimed at assessing the determinants of the availability of HIV Tracer Commodities in Health Facilities in Wakiso District, Uganda.

**Methods:**

A descriptive cross-sectional design was conducted in 42 Health Facilities [HFs] offering HIV/AIDs services in Wakiso District, Uganda. Semi-structured questionnaire adapted from the Anti-Retroviral Therapy Supervision Performance and Recognition Strategy [ART SPARS] tool Version 2.0 | 2018111 was used to collect data.

**Results:**

The majority of the HFs 28 [67%] had all the seven tracer commodities on the day of the visit. The majority of the HFs 33 [78.6%] were using Manual stock management tools that were fully updated. The availability of HIV tracer commodities was high in facilities that made timely ordering [AOR: 2.538, 95% CI: 2.126–3.304, *p*-value = 0.003] while the use of manual LMIS alone at the facility [AOR: 0.623, 95% CI: 0.131–0.958, *p*-value = 0.002] was associated with low availability.

**Conclusion:**

This study indicated that 67% of health facilities visited had all HIV Tracer commodities on the day of the visit. ART commodity management should be computerised and orders made on time to ensure the availability of commodities.

## Introduction

Globally, HIV remains a major public health concern though several gains have been made towards eliminating it in the last two decades. According to the 2023 UNAIDS report, 37.5 million [31.8 million–43.6 million] of the 39 million [33.1 million–45.7 million] people living with HIV [PLHIV] worldwide in 2022 were adults aged 15 years and older 70% of whom reside in sub-Saharan Africa (Musekiwa et al., 2022). The 2022 Uganda Population-based HIV Impact Assessment [UPHIA] findings highlight that Uganda has a 5.8 per cent HIV prevalence rate among adults between the ages of 15 and 49 (Uganda AIDS Commission, 2022)

As the world races to achieve the 95-95-95 goal to end the HIV pandemic by 2030, the number of patients receiving antiretroviral Therapy [ART] has increased through the years (Muyingo et al., 2022). It is critical that the health supply chain is managed effectively and efficiently to ensure the availability of commodities (Lugada, Ochola et al., 2022) especially those that manage HIV/AIDS. This is to avoid the risk of treatment interruption, antiretroviral resistance, treatment failure, morbidity and mortality due to stock-out of HIV-related commodities (Gils et al., 2018). In addition, supply disruptions of other HIV commodities, such as diagnostic tests, cause gaps in testing activities and delays in treatment initiation, while stock-outs of commodities for prophylaxis, such as Cotrimoxazole, interfere with the successful prevention of opportunistic infections (Berhanemeskel et al., 2016).

The stock-outs of HIV commodities and related supply chain management bottlenecks are common in sub-Saharan Africa (Schneider et al., 2006). For instance, almost 36% of facilities assessed in the reporting year 2016 experienced a stock-out falling short of the zero ARV drug stock-out recommendation by the World Health Organization [WHO] (WHO, 2016). Despite the donor support in Uganda, stock-out of antiretroviral drugs at point-of-care is still common a situation that has been further exacerbated by the implementation of universal ‘test and treat’ which commenced in 2017 and has led to a dramatic increase in new ART enrolments (Zakumumpa et al., 2017).

If viral suppression is to be achieved, an uninterrupted supply of Antiretroviral [ARVs] is required. The determinants of stock availability have been previously highlighted in a study conducted in Ethiopia to include, order fill rate challenges, lead time and refill of near expiry commodities, job dissatisfaction among supply chain staff, supply chain management training gaps, improper use of health and logistics management information system (Damtie et al., 2020). In the Ugandan context, little is known about the determinants of stock availability of ARVs.

While many studies have been undertaken on health supply chain systems to assess current challenges, structure, performance, and implications for system strengthening in Uganda's health supply chain (Lugada et al., 2022), literature about the availability of HIV-related commodities and factors associated are scarce. Consequently, this study sought to assess the determinants of stock availability of HIV-related commodities to guide on how improvements can be made to ensure commodity availability at all times.

## Methods

### Study design and setting

This was a facility-based cross-sectional study to assess the determinants of availability of HIV Tracer Commodities in Health Facilities in Wakiso District, Uganda. The study employed quantitative data collection method. Wakiso District is in the Central Region of Uganda that partly encircles Kampala, Uganda's capital city. The prevalence of HIV in Wakiso district is 8% against the national average of 6%. Wakiso has 66 facilities actively offering ART services.

### Study population and sampling

This study was conducted in Wakiso district facilities accredited to offer HIV services. A census approach was used and all facilities (66) in the district actively offering HIV services were considered in this study. However, nine facilities were excluded because (1) four were Centres of Excellence/Specialised facilities with special characteristics that would bias actual findings; (2) two facilities were attached to the military, and two to prisons and these needed more approvals and authorisation beyond clearances from Institutional Review Board [IRB] and these were very difficult to get given the pre-defined data collection period; and (3) one was an Island facility- Bussi HC III, thus, was a hard to reach facility. Thus, 57 health facilities were involved in this study [4 Health Centre II, 34 Health Centre III, 9 Health Centre IVs, 9 Hospitals and 1 regional referral hospital]. Twenty eight of these were pubic facilities, 19 Private not for Profit [PNFPs] and 10 Private for Profit [PFPs].

### Data collection instruments and measurements

Data were collected using a semi-structured questionnaire. This questionnaire was adapted from the standardised and validated Anti-Retroviral Therapy Supervision Performance and Recognition Strategy [ART SPARS] tool Version 2.0. 2018111 http://library.health.go.ug/sites/default/files/resources/ART%20SPARS%20Supervision%20Data%20collection%20tool%2020181112.

This is a multi-pronged intervention strategy tool that assesses the performance of health facilities in areas of rational medicine use, stock and storage management, and ordering and reporting to identify and prioritise problems and measure progress with recognition for improved performance linked to certification for Good Pharmacy Practices. The adapted tool was pretested in Kiruddu National Referral Hospital, Kampala. The dependent variable was the availability of HIV tracer commodities. HIV commodity tracer availability was considered as the availability of all seven tracer commodities as noted below:

*[Abacavir/Lamivudine[ABC/3TC]120/60 mg, Dolutegravir10mg [DTG], Tenofovir/Lamivudine/Dolutegravir300/300/50 mg [TDF/3TC/DTG], Cotrimoxazole 960 mg, Nevirapine [NVP] 10 mg/ml oral suspension, Determine HIV ½, HIV 1/2 Stat Pak]* in the stores on the day of the visit. Overall availability was obtained by dividing the number of facilities with all the seven tracer commodities by the total number of facilities in the study. Months of stock were determined by dividing stock on hand by total consumption in three months factoring in days out of stock. The stock card was the source document for these data.

The independent variables were health facility characteristics [Level of care, facility ownership, number of patients under care, years of provision of ART services], stock management practices [expiries monitoring, stock-outs, consumption rate], warehouse practices, ordering and reporting, and human resource capacity.

Stock levels were determined by categorising a commodity being over-stocked when it has more than 4 months of stock, adequately stocked when having two to four months of stock, under-stocked when less than two but greater than zero months of stock. Stock-out was the absence of at least one tracer commodity on the day of visit from stores.

### Data collection procedures

The data were collected by three [3] research assistants with knowledge on inventory management, the research assistants were trained for two [2] days on data collection tools and source documents. The data collection tool was pre-tested before the actual data collection. The health facility characteristics were provided by the Facility In Charge. Stock management practices were assessed by reviewing the Logistic Management Information Systems [LMIS] on whether they were electronic or manual and on whether they were routinely updated. Commodity traceability was determined by assessing the availability of transaction documents such as Delivery Notes, Issue Requisition vouchers and their routine usage.

Store management practices were determined by assessing the arrangement of commodities, availability of shelves and temperature monitoring practices. Human resources data were obtained by interviewing the stores in-charge on the availability of stores persons, their level of education and their supply chain management-related training. To understand the Ordering and Reporting disparities of health facilities, copies of orders generated and submitted within the review period were assessed for their timeliness, correctness and completeness. Warehouse Performance was assessed by reviewing compliance to delivery schedules and order fill rates of the items ordered.

Expiries monitoring as a determinant of stock availability was determined by assessing expiries monitoring practices of the facilities, availability of expiries at the facility on the day of visit and disposal practices.

### Data analysis

The data analysis was done using SPSS version 26. A descriptive analysis was conducted to determine the frequencies and proportions of the different variables including health facility characteristics, stock availability, stock levels, stock management practices, commodity traceability, stores management, human resource, ordering and reporting, warehousing and expiries monitoring disparities at different facilities. Bivariate and multivariate logistic regression analyses were used to compute the associations between the HIV tracer commodity availability and independent variables. Variables with *P*-value less than 0.05 at 95% confidence interval were considered statistically significant.

## Results

### Health facility characteristics

A total of 42 out of 57 health facilities participated in the study giving response rate of 72%. Data from 15 facilities could not be obtained either because it was unavailable or facilities were unwilling to participate in the study on the day of visit. All facilities 42[100%] were providing Testing, Paediatric, Adult and PMTCT services. Twenty seven, 64.3%, were Health Centre [HC] II/IIIs, 21.4% [ 9] were HC IVs and 14.3%[ 6] were General Hospitals [GH]. Two third, 66.7% [28], of the facilities assessed were public facilities, 23.8% [10] were Private for Profit [PFP] while 9.6% [4] of the facilities where Private Not for Profit [PNFPs]. Seventeen [40.5%] were high-volume facilities [>1000 patients] while 25 [59.5%] were low-volume facilities [<1000]. Table 1 gives more details.

**Table 1.** Showing the health facility characteristic.

Variables	Frequency *N* = 42	Percentage %
ART services provided		
All ART services [Testing, Paediatric, Adult, PMTCT]	42	100
Facility Level of Care		
General Hospital	6	14.3
Health Centre IV	9	21.4
Health Centre II/HC III	27	64.3
Facility Ownership		
Private Not For Profit	4	9.6
Private For Profit	10	23.8
Government	28	66.7
Number of Patients in ART Clinic		
High volume [≥1000]	17	40.5
Low volume [<1000]	25	59.5
Period of Provision of ART Services		
<5 years	4	9.5
≥5 years	38	90.5
Supplying Warehouse		
Joint Medical Stores	14	33.3
National Medical Stores	28	66.7

### Assessing stock availability

**Figure 1.**
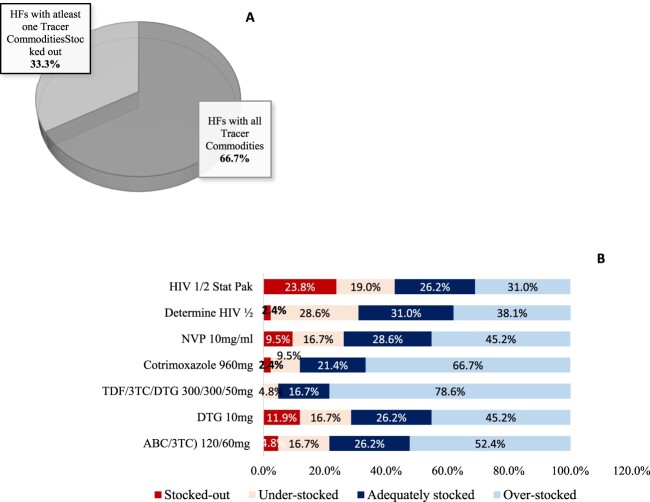
(A): Overall availability of Tracer Commodities, (B): Tracer Commodity Stock Levels at the facilities.

Overall, 28 health facilities [66.7%] had all the seven tracer commodities available on the day of visit (Figure 1(A)). All health facilities [100%] had *Tenofovir/Lamivudine/Dolutegravir [TDF/3TC/DTG 300/300/50 mg]* available while Stat pak was available in 76% of the health facilities. Figure 1(B) shows that TDF/3TC/DTG 300/300/50 mg was the most overstocked commodity in all facilities at 85.7%. All PNFP facilities had all tracer commodities available while only 60% of the PFPs had the commodities available. It was observed that 70.4% of the HC II and HC III had all Tracer commodities compared to 66.7% of HC IV and 50% of GH.

### Availability and utilisation of LMIS tools

Of the Health Facilities accessed, 33[78.6%] had only manual LMIS while 9 [21.4%] facilities had both Manual and Electronic LMIS. All the LMIS were updated routinely and physical stock counts conducted and recorded at least bimonthly.

All 42[100%] of the health facilities had Delivery Notes on site for the review period with Issue/Requisition Vouchers available and filled in with commodities arranged in systematic orders, with no commodities on the floor and had storage temperature routine monitoring. All 42[100%] health facilities had dedicated logistic management staff, of those, 26[61.9%] of the facilities, the logistic management staff were nurses previously trained in inventory management.

Most 41[97.6%] of the health facilities had copies of ARVs submitted orders and all 42[100%] had copies of the test kits submitted copies though only 73.8% were taking orders on time to the delivery schedule and more than half [59.5%] had late deliveries from the supplying warehouse within the review period.

### Determinants of HIV tracer commodities

Table 2 highlights that the availability of HIV commodities is significantly associated with the existing LMIS system and timeliness of ordering for the commodities. Availability of HIV commodities at the health facilities was low among the health facilities who had only manual LMIS system compared to those that had both manual and electronic LMIS systems [COR: 0.543, 95% CI: 0.120–0.960, *p*-value = 0.029].

**Table 2.** Bivariate and multivariate analysis of determinants of availability of HIV commodities at Health facilities offering HIV services.

Variables	COR [95% CI]	*p*-value	AOR [95% CI]	*p*-value
Facility Level of Care				
Health Centre II/HC III	0.421 [0.070–2.550]	0.347	0.300 [0.100–1.089]	0.770
Health Centre IV	0.500 [0.060–4.153]	0.521		
General Hospital	1.0		1.0	
Number of Patients in ART Clinic				
Low volume [<1000]	1.350 [0.359–5.078]	0.657	0.330 [0.290–1.997]	0.150
High volume [≥1000]	1.0		1.0	
Period of Provision of ART Services				
<5 years	2.167 [0.272–7.272]	0.465	2.187 [0.531–3.753]	0.101
≥5 years	1.0		1.0	
Supplying Warehouse				
Joint Medical Stores-JMS	0.720 [0.179–2.901]	0.644	0.641 [0.401–2.147]	0.411
National Medical Stores-NMS	1.0		1.0	
Existing LMIS System				
Manual	0.543 [0.120–0.960]	**0.029**	0.627 [0.132–0.968]	**0.002**
Electronic and Manual	1.0		1.0	
Stores Staff Qualification				
Nurse	0.764 [0.750–4.898]	0.764	0.437 [0.158–3.279]	0.420
Medical records Officer	5.000 [0.584–9.797]	0.142	7.673 [0.725–8.256]	0.191
Others	1.0			
Timelines of orders				
Timely ordering	2.037 [1.806–5.147]	**0.025**	2.545 [2.126–3.304]	**0.003**
Late ordering	1.0		1.0	
Timeliness of delivery				
Timely deliveries	0.462 [0.116–1.829]	0.271	0.64 [0.253–1.654]	0.720
Late deliveries	1.0		1.0	

Note: The significance of bold values represent timely ordering and use of both manual and electronic LMIS.

High availability of HIV tracer commodities was reported in health facilities that ordered on time compared to those that had late order submissions [COR: 2.037, 95% CI: 1.806–5.147, *p*-value = 0.025]. On adjustment of variables from the bivariate level analysis, availability of HIV commodities at the health facilities was low among the health facilities who had only manual LMIS systems compared to those that had both electronic and manual LMIS systems [AOR: 0.623, 95% CI: 0.131–0.958, *p*-value = 0.002]. While high availability of HIV commodities was reported in health facilities which had timely ordering within the cycle as compared to those who had late order submission [AOR: 2.538, 95% CI: 2.126–3.304, *p*-value = 0.003].

## Discussion

Commodity availability at all times is critical if we are to achieve timely and quality HIV services a major indicator in achieving the elimination of HIV by 2030. The current study highlights the availability of HIV-related commodities and its determinants. This is important as it provides a better understanding of the determinants of HIV commodities across health facilities to inform and guide policies and practices aimed at ensuring zero stock-outs of commodities to minimise care interruption among HIV patients. This will ultimately contribute to the attainment of the goal of national medicine policies of ensuring access to HIV commodities and the 95-95-95 global HIV eradication target.

### Stock availability of HIV commodities

The study findings indicated that overall, 66.7%, of HFs had all the HIV tracer commodities, on the day of the visit with 100% of all PNFP facilities having all the seven tracer commodities. The current findings are slightly lower than those reported in a study done in Ethiopia where they found availability of commodities was 72.75%[15]. However, these findings are higher than those reported in a study done in Kinshasa on stock-outs of HIV commodities in public health facilities where availability was witnessed at 37% [11/41 facilities] (Gils et al., 2018). Stock-outs in facilities of vital HIV items affect the access to vital commodities; ultimately, there is a likelihood of increased viral resistance and increased HIV transmission risk leading to increased morbidity and mortality, especially in individuals with poor immunity (Mori & Owenya, 2014).

Stock-outs were witnessed with Nevirapine [NVP] 10 mg/ml oral suspension a commodity used in the prevention of mother-to-child transmission in 9.5% of the facilities, while Stat pak [Test Kit] stock-outs were witnessed in 23.8% of the facilities similar situation was reported in Ethiopia (Damtie et al., 2020). Besides, a study conducted in Uganda, Rwenzori Region on managing stock levels of HIV commodities using electronic systems highlighted that Stat pak was the most stocked-out testing kit among kits reviewed (Mori & Owenya, 2014) . These similarities may be because both studies were conducted in Uganda where similar supply chain practices are observed across. The stock-out of Nevirapine [NVP] 10 mg/ml oral suspension is used for prophylaxis as a main intervention in the prevention of mother-to-child transmission of HIV-1 (Napyo et al., 2020) the absence of which increases chances of transmission. On the hand stock-out Stat pak [Test Kit] implies that testing cannot be done efficiently. Scaling up HIV testing is an important strategy in identifying unknown positives and preventing the onward transmission of HIV (Kharsany & Karim, 2016).

### Determinants of stock availability

Up to 26.2% of the facilities had at least ordered for commodities late and 59.5% of the facilities had at least ever received a late delivery within the review period. Timeliness of ordering was significantly associated with the availability of HIV commodities at the facilities. The current findings are similar to a study conducted in Uganda and Saudi Arabia (AlRuthia et al., 2023; Lugada et al., 2022) which revealed that the main reasons for stock-outs in facilities were discrepancies in orders and deliveries. The current findings may be due to poor inventory management practices and wrong LMIS systems (AlRuthia et al., 2023; Berhanemeskel et al., 2016). Discrepancies in the ordering of commodities as seen in this study affect commodity availability contributing to stock-outs a major parameter in limiting the accessibility of vital HIV commodities.

The study found that use of an electronic LMIS system was positively associated with the availability of HIV commodities; similar findings were seen in the study on the effects of Information and Communication Technology on health service delivery at Tafo Government hospital in Ghana that highlighted electronic records being essential for improving the quality of healthcare delivery and improving efficiency (Kabanda et al., 2019). The role of the electronic system is further highlighted in a study on leveraging electronic logistics management information systems to enhance and optimise supply chain response during public health emergencies during the COVID-19 response in Uganda that enabled the Ministry of Health track the distribution of medical countermeasures through the warehouses, eight regional pre-positioning centres, and over 2000 user units in 136 districts (Napyo et al., 2020). These findings may be because electronic systems offer quality data, thus leading to reduced errors (Menachemi & Collum, 2011) when generating commodity orders, for instance. Having an electronic LMIS system may enhance the timely availability of data, hence contributing to greater commodity and better health outcomes (Lugada, Komakech et al., 2022).

Although the current findings did not statistically support the association between the tracer commodity availability and facility level of care, the study about supply chain performance conducted in Uganda observed lower stock-outs in lower-level health facilities compared to higher-level facilities (Lugada, Ochola et al., 2022). This is perhaps due to patients bypassing lower-level facilities for higher-level facilities perceived to have better care (Kharsany & Karim, 2016).

### Strengths and limitation

This study reviewed primary data straight from the inventory management tools such as stock cards, ordering forms which reduced chances of error seen in when secondary data are used. However, the desired number of facilities was not attained that could have affected the significance of some variables. Still, the 72% response rate attained was within the acceptable range. Stock availability was only assessed on the day of visit and the study didn’t go into understanding availability patterns beyond the visit day. Further studies are required to explore poor quantification practices and central warehouse Procurements and their effect on commodity availability.

## Conclusion

The study found out that one third of the HFs had at least stocked out of one tracer item and this was more pronounced in private health facilities. Timely ordering from health facilities, use of electronic systems, and efficient warehouse performance were the determinants that influence the availability of HIV Tracer Commodities a major parameter in achieving the 95-95-95 global targets.

## Data Availability

The datasets used and/or analysed during the current study are available upon reasonable request.
